# Early Detection of Alzheimer’s Disease via Machine Learning-Based Microwave Sensing: An Experimental Validation

**DOI:** 10.3390/s25092718

**Published:** 2025-04-25

**Authors:** Leonardo Cardinali, Valeria Mariano, David O. Rodriguez-Duarte, Jorge A. Tobón Vasquez, Rosa Scapaticci, Lorenzo Crocco, Francesca Vipiana

**Affiliations:** 1 Department of Electronics and Telecommunications, Politecnico di Torino, 10129 Torino, Italy; leonardo.cardinali@polito.it (L.C.); valeria_mariano@polito.it (V.M.); david.rodriguez@polito.it (D.O.R.-D.); jorge.tobon@polito.it (J.A.T.V.); 2 Institute for the Electromagnetic Sensing of the Environment (IREA), National Research Council of Italy, 80124 Naples, Italy; scapaticci.r@irea.cnr.it (R.S.); crocco.l@irea.cnr.it (L.C.)

**Keywords:** Alzheimer’s disease, classification algorithms, head phantom, machine learning, microwave antennas, microwave sensing, multilayer perceptron

## Abstract

The early diagnosis of Alzheimer’s disease remains an unmet medical need due to the cost and invasiveness of current methods. Early detection would ensure a higher quality of life for patients, enabling timely and suitable treatment. We investigate microwave sensing for low-cost, non-intrusive early detection and assessment of Alzheimer’s disease. This study is based on the emerging evidence that the electromagnetic properties of cerebrospinal fluid are affected by abnormal concentrations of proteins recognized as early-stage biomarkers. We design a conformal six-element antenna array placed on the upper portion of the head, operating in the 500 MHz to 6.5 GHz band. It measures scattering response due to changes in the dielectric properties of intracranial cerebrospinal fluid. A multi-layer perceptron network extracts the diagnostic information. Data classification consists of two steps: binary classification to identify the disease presence and multi-class classification to evaluate its stage. The algorithm is trained and validated through controlled experiments mimicking various pathological severities with an anthropomorphic multi-tissue head phantom. Results support the feasibility of the proposed method using only amplitude data and lay the foundation for more extensive studies on microwave sensing for early Alzheimer’s detection.

## 1. Introduction

Alzheimer’s disease (AD) affects millions of people each year, especially those over 65. It is marked by a decline in cognitive functions, including memory loss, difficulties with language, spatial awareness, face recognition, and reasoning [1]. The exact causes of AD are yet to be clearly understood. Still, there is a correlation with factors such as familiarity, high blood pressure, smoking, excessive alcohol consumption, physical inactivity, social isolation, and depression [2].

There is no currently available single test capable of definitively diagnosing Alzheimer’s disease or other forms of dementia. Instead, physicians rely on a combination of diagnostic tools, including neurological and behavioral examinations, cognitive and functional tests, genetic testing, brain-imaging techniques such as magnetic resonance imaging (MRI), computed tomography (CT), and positron emission tomography (PET), and analyses of cerebrospinal fluid (CSF) or blood biomarkers. These tools are integrated with the patient’s medical history and other relevant information to ensure the most accurate diagnosis [3,4,5].

With the progress of the disease, at the middle- and late stages, amyloid plaques and neurofibrillary tangles accumulate in the brain, followed by brain atrophy. MRI, PET, and CT can detect these AD biomarkers [6,7]. However, these techniques are most effective at the latter stages of the disease. Moreover, they are expensive, require bulky equipment, and may involve ionizing radiation exposure in the case of PET and CT.

Research shows that AD induces physiological changes up to twenty years before symptoms become evident [8]. The first indicators of AD are elevated concentrations of amyloid-beta $A\beta_{1–40}$, tau, and p-tau, and the increasing amyloid-beta $A\beta_{1–40}\;/A\beta_{1–42}$ ratio in the CSF [9,10,11]. Amyloid-beta and tau proteins form amyloid plaques and neurofibrillary tangles, respectively, which are known to contribute to brain degeneration in AD. Yet, estimating those biomarkers is not trivial. The standard methodology analyzes a CSF sampled through a lumbar puncture extraction, a highly invasive procedure that often causes pain and discomfort. Ongoing research aims to identify these biomarkers in blood and urine and evaluate their reliability as predictive AD indicators. Alternative early diagnostics include microbiota-based methods and genetic tests. Machine learning (ML) can integrate these techniques to improve accuracy. In [12,13,14,15,16,17], ML algorithms have been explored to analyze speech data as a potential diagnostic tool. Overall, there is no established technique for early AD detection, and the interest in non-invasive, low-cost, portable alternatives is open. These would enable access to medical treatment before the disease symptoms become evident—i.e., at a stage of the disease when current treatments are more effective.

In [18,19], a correlation has been shown between the concentration of $A\beta_{1–42}$ and $A\beta_{1–40}$ in the CSF and its electromagnetic properties (i.e., dielectric relative permittivity and electrical conductivity). This led us to propose identifying these permittivity alterations due to $A\beta_{1–42}$ and $A\beta_{1–40}$ concentration variations through microwave sensing (MWS) technology and ML algorithms [20,21,22]. Moreover, the chiral properties of Aβ have been numerically investigated, exploring the consequent deflection of incident electromagnetic waves [23]. MWS is a non-invasive, low-cost method that investigates dielectric properties through interactions with electromagnetic fields [24]. Raw MWS data can be complex, noisy, and difficult to interpret. Machine learning (ML) enhances MWS capabilities by improving data processing, pattern recognition, and decision-making [25].

MWS is used in applications where variations in dielectric contrast leave a measurable impact on the system’s scattering response. This principle is leveraged in various fields, including structural damage analysis [26,27], food quality assessment [28,29], stroke detection [30,31,32], glucose sensing [33,34], and breast cancer screening [35,36,37,38]. Recently, MWS has also been investigated for neurodegenerative disorders, including AD [39,40,41,42,43]. In [39], a wearable device equipped with electromagnetic sensors was designed to non-invasively track brain atrophy and the expansion of the lateral ventricles associated with Alzheimer’s disease, performing numerical and experimental validation. Numerical studies on MWS have been conducted in [40,41,42] to detect different stages of Alzheimer’s disease by analyzing dielectric changes in gray and white matter as well as brain atrophy. In particular, Refs. [40,41,43] utilize machine learning algorithms to classify data samples according to disease severity.

To the best of our knowledge, this is the first experimental demonstration of using ML-based MWS to identify and classify CSF permittivity changes linked to early AD biomarkers. To this end, starting from numerical feasibility studies and preliminary assessment of the approach mechanism [20,21,22], this work recreates realistically different pathological AD severity scenarios with an ad-hoc anthropomorphic multi-tissue head phantom, and a six-antenna-based MWS composed of a multi-port vector network analyzer (VNA) and custom monopoles featuring a simple design. The antennas are designed to operate from 500 MHz to 6.5 GHz, considering adequate sensing penetration, system dynamics, and scattering sensitivity. A multilayer perceptron (MLP) is then optimized to classify CSF conditions across different severity levels using measured scattering parameters. Multiple training, validation, and testing schemes support the reliability of the results. The analysis is carried out using both complex and only amplitude scattering parameters, showing comparable performance, with over 94% accuracy in detection and an f1-score of up to 87% in severity classification.

The structure of this paper is as follows: Section 2 describes the implemented MWS system. Section 3 details the experimental validation, including the phantom creation and measurement procedure. Section 4 provides a comprehensive description of the machine learning algorithm used for classification. The results of the testing procedure on the test set are presented in Section 5, and finally, Section 6 summarizes the conclusions of this study.

## 2. Microwave Sensing System

**Figure 1:**
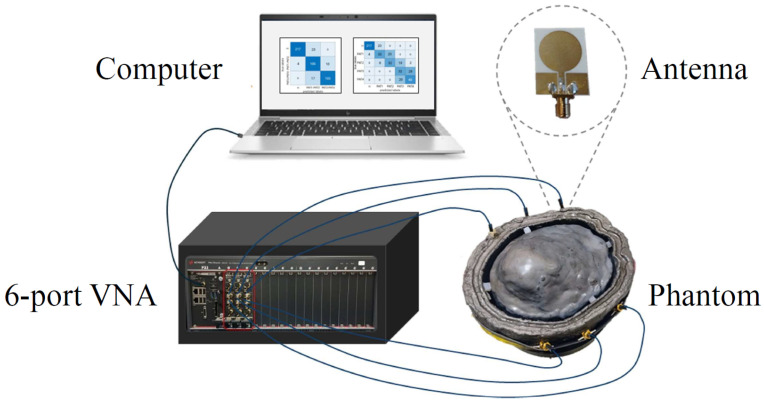
Microwave sensing system.

The implemented MWS system, depicted in Figure 1, consists of a laptop and an M9804A PXIe six-port VNA (Keysight Technologies, Santa Rosa, CA, USA) [44], connected via low-loss coaxial cables to a custom wideband six-antenna array. The antennas are conformally positioned along the sides of the upper portion of a head phantom. The VNA acquires the scattering parameters through the antenna array and sends the measured data to the laptop for processing. The antennas are placed on the lateral sides of the head phantom rather than the front and back, as the relatively thinner skull in these regions facilitates deeper field penetration.

**Figure 2:**
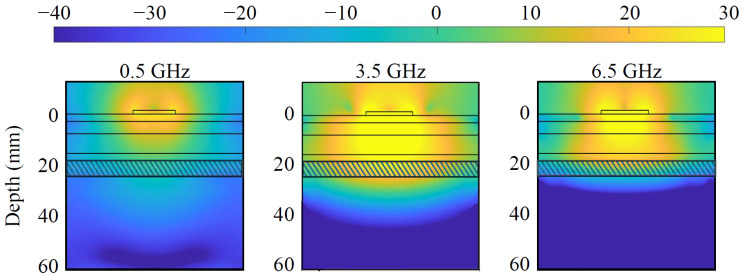
Simulated power density distribution [dBW/$m^{2}$] within the multi-tissue block at 0.5, 3.5, and 6.5 GHz. The various tissues are represented by layers, with the striped layer indicating the CSF.

Various antenna designs have been proposed in the literature for human body monitoring using microwave technologies, e.g., [45,46,47]. In our work, each antenna of the sensing system is a circular monopole antenna printed on Rogers RO4003C substrate (Rogers Corporation, Chandler, AZ, USA) by PCB international (Seattle, WA, USA) with a standard thickness of 1.52 mm, entailing easy manufacturing and compactness. The working frequency band of the antenna is about 500 MHz–6.5 GHz, in agreement with the device requirements. The minimum frequency is dictated by the thickness of the CSF layer, which corresponds to around $\lambda_{\mathrm{CSF}}/10$ where $\lambda_{\mathrm{CSF}}$ is the wavelength inside the CSF. The highest frequency is chosen according to the wave penetration within the head. Considering the variability of the human head, the antenna is optimized by placing it on a multilayer block of size 100×100×63 ${\mathrm{mm}}^{3}$, a simplified scenario that mimics the head’s multi-layer structure and dielectric properties. The block is constituted of six stacked slabs, encompassing skin, fat, skull, fat, CSF, and gray matter, with thicknesses of 3, 5, 8, 3, 6, and 38 mm, respectively, as portrayed by the contour lines in Figure 2, and dielectric characteristics taken from [48].

**Figure 3:**
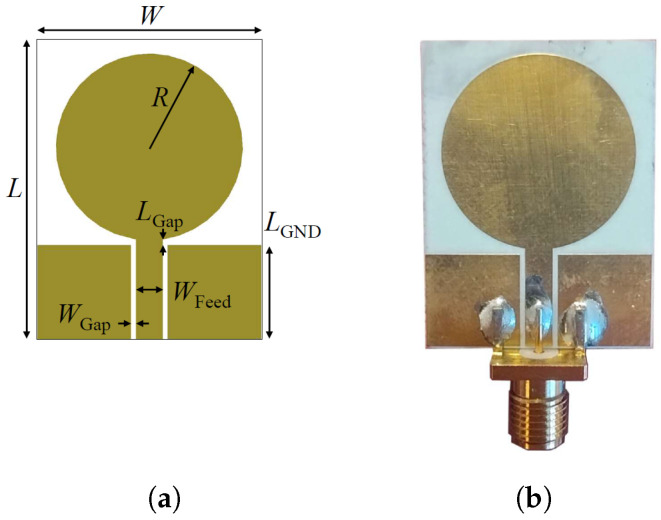
Circular monopole antenna: (**a**) geometry of the antenna, (**b**) realized antenna.

Figure 2 depicts the simulated power density distribution within the multi-tissue block at the lowest, center, and highest frequencies. A lower power density can be observed at the lowest frequency, 500 MHz, due to the non-optimal antenna matching and decreased penetration at the highest, 6.5 GHz, due to higher tissue losses. Nevertheless, the power density distribution reaches an adequate level at CSF in each case, and so does the sensing capability. The antenna optimization uses the circular monopole layout displayed in Figure 3a as a baseline while varying the radius of the circular section (*R*) and the feeding line width ($W_{\mathrm{Feed}}$), maintaining constant the gap ($W_{\mathrm{Gap}}$) between the ground plane and the feeding line and the substrate dimension (W×L). The objective of the optimization is to minimize the reflection coefficients within the desired frequency band. The realized antenna is shown in Figure 3b, with its repetitively optimized geometrical parameters reported in Table 1.

**Figure 4:**
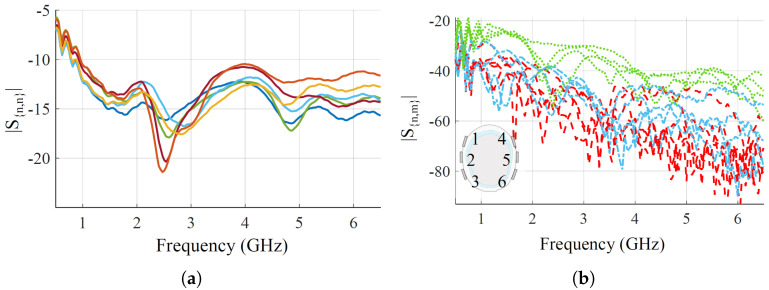
Measured scattering parameters of the six antennas placed on the head phantom. (**a**) Reflection coefficients. Each color represents a different antenna. (**b**) Transmission parameters. The antenna pairs are grouped based on relative distances: close pairs are indicated by green dotted lines, medium-distance pairs by blue dashed lines, and long-distance pairs by red dashed lines. A scheme of the antenna positions on the phantom in shown in the corner.

Finally, Figure 4 shows the measured reflection and transmission coefficients, when placing the optimized antennas on the head phantom. Regarding the reflections, all the antennas have very good matching, lower than −10 dB, from around 1 GHz up to the end of the chosen bandwidth. At the lower end of the bandwidth, from 500 MHz to 1 GHz, the matching is worse but still lower than −5 dB. For reflections, there are above around −90 dB in the whole frequency band for all the antenna pairs, which is within the VNA dynamic range with the chosen setup parameters [44]. The transmission-reported pairs are grouped into three families based on relative distances for visualization. First, close-by pairs, $S_{3,2}$, $S_{2,1}$, $S_{6,5}$, $S_{5,4}$; medium-distance pairs, $S_{3,1}$, $S_{3,6}$, $S_{6,4}$, $S_{4,1}$; and long-distance pairs, the remaining ones.

## 3. Experimental Validation

### 3.1. Ad-hoc Anthropomorphic Head Phantom

**Figure 5:**
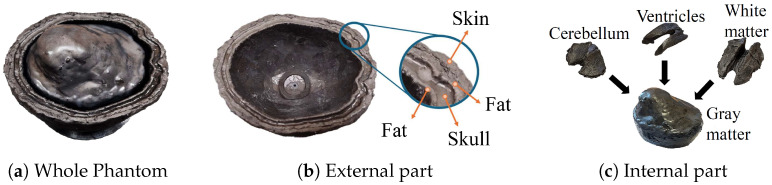
Anthropomorphic multi-tissue head phantom.

This section describes the designed multi-tissue phantom representing a human head. It is used with the MWS system to collect scattering parameters in both healthy and AD conditions. The phantom aims to emulate a human head with all its different tissues while allowing the CSF layer to be changed without interfering with the rest of the setup. The morphology of the different tissues is based on [49]. The phantom includes two concentric parts separated by a 6 mm liquid-filled gap that models the intracranial CSF. This gap is maintained by rubber spacers and a 3D-printed brain support. The external part includes skin, external fat, bone, and internal fat. The internal part represents the brain domain, i.e., white and gray matter, cerebellum, and ventricles. A hole in the bottom of the external part allows draining through a tube, which is clamped when the phantom is filled. Figure 5a shows the assembled phantom, an upside-down human head cut sideways at the “middle nose plane”, that joins the external and internal parts, respectively, shown in Figure 5b,c.

**Figure 6:**
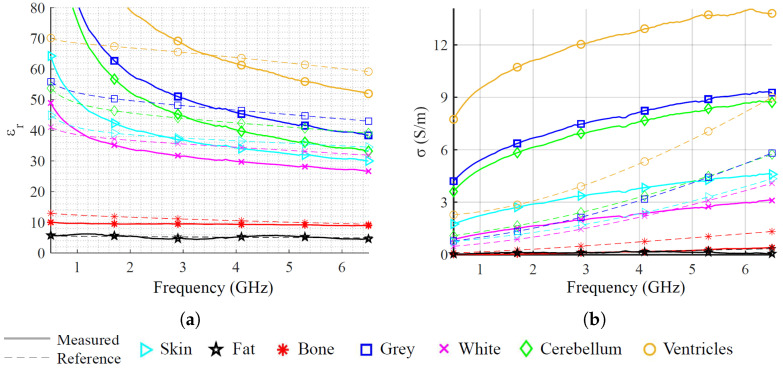
Complex permittivity of the multi-tissue head phantom. (**a**) Relative permittivity. (**b**) Conductivity.

The internal and external fat-mimicking tissues are 3D-printed using carbon-loaded Polylactic Acid (cPLA), which has permittivity and conductivity properties similar to those of human fat. The other tissues, except CSF, are made of platinum-catalyzed silicone rubber mixed with 20 μm diameter graphite powder as detailed in [50]. This approach ensures a simpler and safer manufacturing process, using only two materials. It also avoids toxic substances like carbon black powder, which is used in other phantoms [51], though at the cost of some customization. The weight percentages of graphite for the corresponding mimicked tissues are presented in Table 2, while Figure 6 shows their respective relative permittivities and conductivities. Reference values are from [48]. Measured values are obtained using a Keysigth N1501A dielectric probe (Keysight Technologies, Santa Rosa, CA, USA), the Keysight N1500a software version 20.0.24092501 [52], and the method in [50].

Then, considering the dispersiveness and losses of the brain tissues and the intrinsic freedom-degree of customization of the rubber–graphite mixtures, we prioritized the relative permittivity at the central frequency, 3.5 GHz. This yelded better agreement with reference values both at and above this frequency as shown in Figure 6a. For the conductivities (Figure 6b), the reached values overestimate the reference values, which is a worse case due to the higher attenuation of the propagating waves. Despite the limitation of a perfect agreement with the reference values, we can consider the overall phantom suitable for the proofing-of-concept of an ML-driven MWS since it is representative of the complexity and challenges of an anthropomorphic multi-tissue condition.

**Figure 7:**
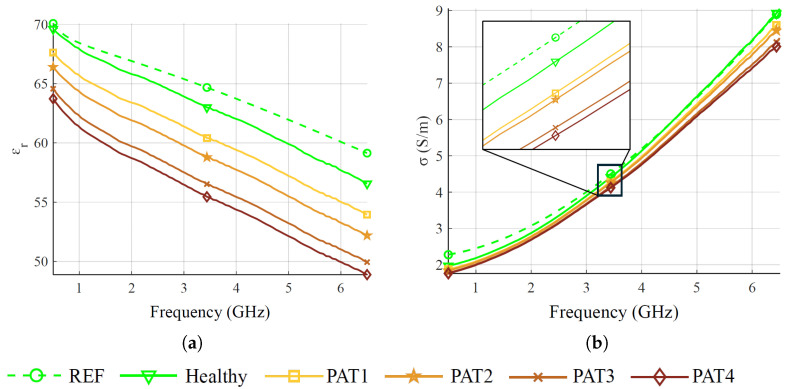
Measured permittivity of CSF at different pathological severity. (**a**) Relative permittivity. (**b**) Conductivity. REF indicates the reference values taken from the CNR-IFAC database [48].

The CSF is produced using water, salt, and triton X-100 mixtures, as in [53]. One mixture is produced to simulate the healthy condition, and four to mimic the pathological condition at different severity grades, corresponding to four permittivity reductions, respectively. Permittivity reduction is based on [19], which reports a 5% drop in CSF permittivity at 1 GHz in pathological cases. Figure 7 shows the permittivity for the four emulated CSF pathological stages, dubbed PAT1:4, indicating, respectively, reductions of 2.5%, 5%, 7.5%, and 10% relative to the healthy scenario at 1 GHz. However, variations in the relative permittivity are almost constant in the whole considered band. The specific composition of each fluid is detailed in Table 3.

### 3.2. Measurement Procedure

During the measurement of the scattering parameters, a Multiple Input Multiple Output (MIMO) configuration is employed. Both transmission and reflection parameters are considered to fully characterize the system’s response.

The measurement procedure follows a repetitive three-step cycle: first, filling the gap in the head phantom with the corresponding CSF liquid; second, acquiring the scattering parameters; and third, draining the liquid from the phantom. Then, the acquisition procedure repeats the cycle while alternating the healthy CSF with the pathological variants, which are randomly poured into the phantom to avoid systematic biases, resulting in four healthy and four pathological scenarios. To build the dataset, we conducted measurements over three days: six acquisitions on the first two days, and three on the last.

A measure consists of a 6×6 scattering matrix ranging from 500 MHz to 6.5 GHz, spanned in 101 points, with the VNA set to 0 dBm input power and the IF filter to 100 Hz. Then, each scenario is measured 10 times, taking about 50 s, for 80 measurements per acquisition and a total of 1200 in the whole dataset.

## 4. Machine-Learning-Driven Sensing

This work employs the MLP algorithm, a type of Artificial Neural Network (ANN) commonly used for complex medical classification tasks [54]. For instance, it has been applied in [55] for brain stroke detection, and in [56] for the detection and monitoring of heart and liver diseases and for lung cancer. The MLP is based on a number of neurons, as in the human brain, which are linked together with weighted connections, and the output of each neuron is regulated by an activation function. The network consists of at least three layers of neurons: an input layer, a hidden layer, and an output layer [57]. The supervised method is adopted, where the algorithm is trained on labeled samples to create a surrogate model. In this study, the MLP is used for binary classification (healthy vs pathological) and multi-class classification to differentiate AD severity levels.

**Figure 8:**
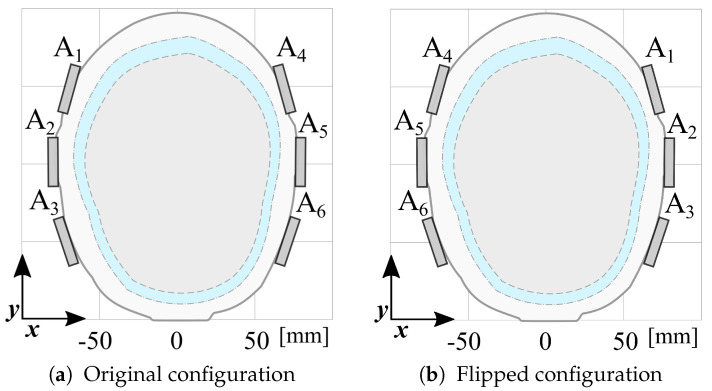
Data augmentation. (**a**) Original array configuration and (**b**) flipped configuration.

The dataset is augmented by doubling its size, generating virtual scattering matrices by swapping left and right antennas, as illustrated in Figure 8. This data augmentation process enhances the variability by accounting for the phantom’s asymmetry and is exploited to overcome the limitations of having a single phantom manufactured for this study.

The augmented dataset used as input for the MLP algorithm is structured as a matrix, where each row represents a measurement, and the columns contain the corresponding reshaped complex scattering parameters at different frequencies, for a total of 6×6×101=3636 columns. Two cases are considered: using real and imaginary parts separately (3636×2=7272 features), or using only the magnitude (3636 features). Considering magnitude-only information may be of utmost importance in practice, due to the lower cost and greater simplicity of receivers that measure the module only [55].

**Figure 9:**
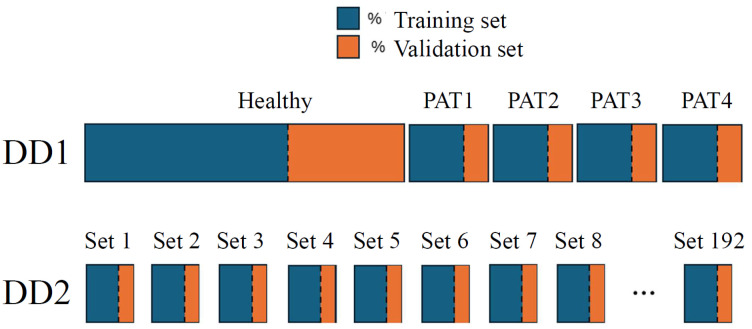
Illustration of the data-splitting process into training and validation sets for DD1 and DD2. The data is randomly divided into training and validation sets using three different proportions: 60:40, 70:30, and 80:20.

The ANN optimization procedure consists of three phases: training, validation, and testing. During training, the model learns patterns and relationships within the dataset by adjusting its parameters to minimize the loss function. The validation tunes hyperparameters and prevents overfitting by evaluating the model’s performance on an independent validation set. Typically, the dataset is divided into training and validation subsets, with a split ratio that ensures sufficient data for learning and model assessment. In this work, the impact on classification performance is evaluated by employing three training–validation split ratios: 60:40, 70:30, and 80:20, using the data from the first two days of measurements. Training and validation sets are generated using two approaches. In data division one (DD1), all the samples are grouped by class. Then, for each class, the samples are randomly split into training and validation sets according to the specified ratios. In data division two (DD2), the 10 measurements within each of the 192 measurement sets are randomly split using the same percentages, ensuring that each measurement set contributes samples to both the training and validation sets; see the scheme in Figure 9. This helps assess whether performance depends on specific measurement sets while maintaining class balance. Finally, the testing phase evaluates the NN’s generalization capability using unseen data, verifying its effectiveness in real-world scenarios. In this case, data from the third day is used.

The hyperparameters affect the model structure and the learning process. In this work, to select the best set of hyperparameters, the grid-search method is used [58]. In this method, different values of the hyperparameters are given as input. For each hyperparameter combination, a model is trained using the training set and then evaluated on the validation set. Finally, the combination with the best score is chosen. For binary classification, the score is evaluated using the accuracy, which measures the proportion of correctly predicted samples as:

(1) $$\mathrm{accuracy}=\frac{\mathrm{TP}+\mathrm{TN}}{\mathrm{TP}+\mathrm{TN}+\mathrm{FP}+\mathrm{FN}}$$

where TP stands for true positive and TN for true negative, indicating the number of samples correctly predicted as positive and negative, respectively. FP refers to false positive, and FN to false negative, indicating the number of samples wrongly predicted as positive and negative, respectively. Moreover, other commonly used metrics for performance evaluation are precision, recall, and f1-score:

(2) $$\mathrm{precision}=\frac{\mathrm{TP}}{\mathrm{TP}+\mathrm{FP}}$$

(3) $$\mathrm{recall}=\frac{\mathrm{TP}}{\mathrm{TP}+\mathrm{FN}}$$

(4) $$\mathrm{f1-score}=2\times \frac{\mathrm{precision}\times \mathrm{recall}}{\mathrm{precision}+\mathrm{recall}}$$

Precision quantifies the proportion of correct positive predictions, while recall measures the proportion of true positive samples correctly identified. The f1-score is the combination of precision and recall via a harmonic mean; it becomes advantageous when the dataset classes are not balanced. It is used as an evaluation score for the multi-class classifier.

During the grid-search optimization process, the considered MLP hyperparameters are: the number of neurons for each layer, the learning rate (the step size towards the minimization of a loss function at each iteration), the training function (the function used to train the algorithm to recognize the input and to produce the correct output), and the loss function. The optimized hyper-parameters for binary classification are reported in Table 4 and Table 5 for DD1 and DD2, respectively, considering the different training and validation proportions and the use of the complex scattering parameters or the module only. Specifically, for the training function, CGB and S-CGB correspond to the conjugate gradient method and scaled conjugate gradient method, while for the loss function, MSE and SAE are the mean squared error and the sum absolute error, respectively.

The other hyperparameters are set, for all the considered configurations, as in [59]: the minimum gradient of the performance function is $10^{-6}$, the validation and test ratios for the training data are 0.15, the momentum (a parameter that helps the training acceleration in the relevant direction and dampens oscillations) is 0.9, and the maximum number of validation failures (the worsening of the performances before training stops) is six. Finally, the number of hidden layers is set to two [20], and the maximum number of epochs to 2000.

## 5. Results and Discussion

In the following, Section 5.1 covers a principal components analysis (PCA). Then, we analyze the proposed system’s performance, investigating first a binary classification in Section 5.2 and then a multi-class classification in Section 5.3.

### 5.1. Principal Components Analysis

To determine whether a simpler classification algorithm, such as a support vector machine (SVM) or decision tree, is suitable for this problem, a PCA is performed on the original dataset, excluding the flipped data. PCA reduces the dimensionality of the dataset by transforming it into a set of uncorrelated principal components, ranked by the amount of variance they capture. Analyzing the explained variance ratio makes it possible to assess whether most of the information is concentrated in a few principal components, indicating that a lower-complexity classifier could effectively separate the data. Additionally, visualizing the data in the space of the first few principal components helps determine whether clear decision boundaries exist, further guiding the choice of an appropriate classification model.

**Figure 10:**
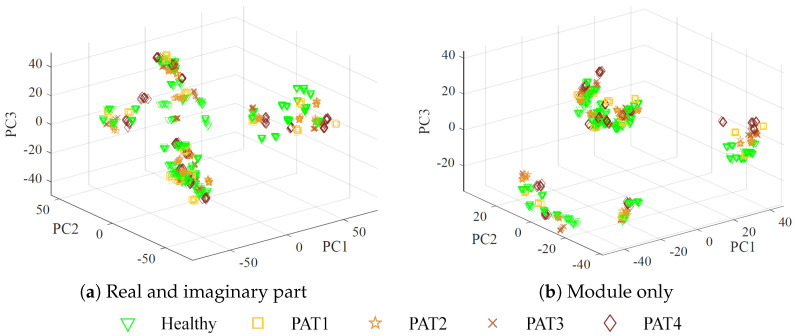
Scatter plot of PCA visualization for the original dataset, with different colors representing the mimicked pathological severity.

Figure 10 presents a scatter plot of the dataset projected onto the three principal components (PC1, PC2, PC3). The plot reveals that the measurements form small clusters, each corresponding to the ten consecutive measurements by a given set. However, these clusters are not perfectly compact due to noise and instrument drift. Additionally, the lack of clear separability using a 3D plane indicates that a more advanced classification algorithm, such as MLP, is required to achieve accurate classification.

### 5.2. Binary Classification

Before discussing the results of the testing phase for the binary classification in detail, Table 6 reports the accuracy values obtained during the validation phase with the optimized hyperparameter configurations. The accuracy reaches high values in all assessed cases, consistently higher than 96.7%.

The results achieved by applying the different trained algorithm models to the testing set, which contains previously unseen data belonging to a different day of measurements, are reported in Table 7 and Table 8 for the DD1 and DD2, respectively (see Section 4). These tables contain the values of accuracy, precision, recall, and f1-score for each test. Each column corresponds to a different training–validation split, as reported in the first row, and the upper part of the tables refers to separate features for the real and imaginary parts of the scattering parameters, while in the lower part, only the module is used.

The algorithm tends to achieve higher performance using the module configuration approach in both the DD1 and DD2; this could be due to inaccuracies in the experimental evaluation of the phase of the scattering parameters, which affect the complex quantities. Regarding the training–validation proportions and the two data divisions (DD1 and DD2), no evident trend emerges: the highest performances with DD1 are obtained using 60%:40% partitioning, and with DD2 using the 70%:30% one.

### 5.3. Multi-Class Classification

Starting from the two previous best-performing binary classifiers (60%:40% for DD1, and 70%:30% for DD2, both considering the module), a further analysis is carried out to assess the AD severity levels. Two possible pathological classifications are considered: first, four classes corresponding to PAT1, PAT2, PAT3, and PAT4, and, second, two classes clustering the two lowest severities, PAT1 and PAT2, and the two highest severities, PAT3 and PAT4. The corresponding optimized MLP hyper-parameters are reported in Table 9. The other hyper-parameters are set to the same values as for the binary classification case (see Section 4).

**Figure 11:**
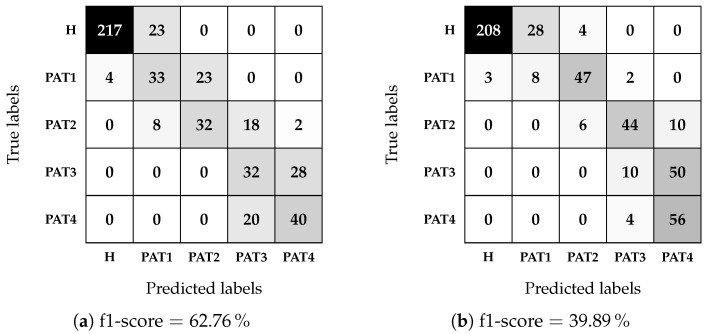
MLP confusion matrices for the multi-class classification with four pathological classes, using the module of the scattering parameters as dataset features. A darker color indicates a higher value. (**a**) DD1, 60:40 (%); (**b**) DD2, 70:30 (%).

**Figure 12:**
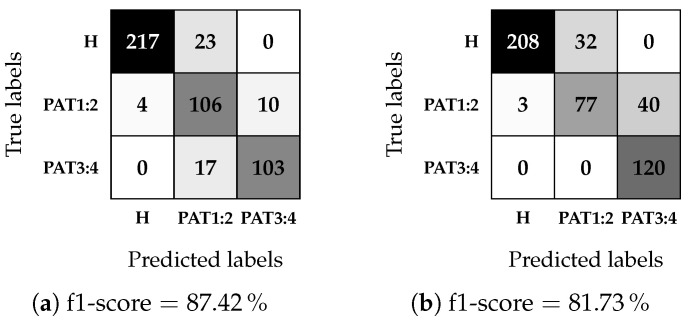
MLP confusion matrices for the multi-class classification with two pathological classes, using the module of the scattering parameters as dataset features. A darker color indicates a higher value. (**a**) DD1, 60:40 (%); (**b**) DD2, 70:30 (%).

The testing phase results are shown as confusion matrices, together with the f1-score, in Figure 11 and Figure 12, where a darker color indicates the closer value to the maximum for the considered-true label: 240 for the healthy case (labeled as H), 60 for each pathological case in Figure 11 (labeled as PAT1, PAT2, PAT3, and PAT4), and 120 for each pathological case in Figure 12 (labeled as PAT1-PAT2 and PAT3-PAT4). It is worth noting that the matrices join the previous binary classification between healthy and pathological cases and the subsequent multi-class pathology classification. The results indicate that the algorithm struggles to distinguish between all four pathological severities accurately but achieves an f1-score of up to 87.42% when considering only two severity levels. Furthermore, the confusion matrices in Figure 11 reveal that misclassifications of pathological cases as healthy CSF predominantly occur in PAT1. The classifier seldom confuses classes with a permittivity difference greater than 2.5%.

## 6. Conclusions and Perspectives

In this work, we described an approach to investigate the feasibility of microwave sensing and machine learning to achieve early AD detection. The physiological basis of this assumption is the reduced permittivity of CSF in AD patients. For this reason, we built a measurement system using a VNA and an array of six custom wide-band antennas designed for this application. To validate the approach, we realized a custom multi-tissue realistic phantom of a human head where the CSF layer can be changed to assume different permittivity values, representing the healthy case or different severity levels of the pathological case. The antennas were placed around the phantom to acquire the scattering parameters in the healthy and AD conditions. A data augmentation technique was used to double the dimension and the variability of the original dataset. Then, the augmented dataset was used to train, validate, and test the MLP classification algorithms. When tested on data belonging to an entirely different day with respect to training and validation, the classifiers achieved an accuracy higher than 94% on binary classification (healthy/pathological), and an f1-score higher than 87% in multi-class classification using two pathological severity levels, confirming the potential impact of this technology on AD early detection.

We acknowledge that real-world scenarios involve additional complexities, and while our proposed method has shown potential, the primary goal of this study is to demonstrate its feasibility. Further research will be necessary to evaluate its effectiveness and generalizability on real patient data. In the future, we plan to conduct controlled in vivo tests to assess the system’s performance in real biological conditions, to combine the designed microwave sensing system with other machine learning algorithms, and to investigate other placements of the antennas to detect the CSF variations. 

## Figures and Tables

**Table 1:** Antenna design parameters.

Label	*W*	*L*	*R*	$W_{\mathrm{Feed}}$	$W_{\mathrm{Gap}}$	$L_{\mathrm{Gap}}$	$L_{\mathrm{GND}}$
Value ^†^	24	32	10	2.9	0.5	0.6	10

^†^ Dimensions in [mm].

**Table 2:** Mass percentage of graphite powder for each tissue.

Tissue	Graphite %
Skin	42
Skull	20
Cerebellum	45
Ventricles	50
Gray matter	47
White matter	41

**Table 3:** Recipes for artificial CSF.

CSF	Water % ^†^	Triton X-100 %	Salt %
Healthy	89.43	9.17	1.40
PAT1 (2.5%) ^‡^	86.86	11.76	1.38
PAT2 (5.0%)	84.96	13.66	1.38
PAT3 (7.5%)	82.99	15.62	1.39
PAT4 (10.0%)	81.95	16.65	1.40

^†^ The percentages refer to mass. ^‡^ Percentage reduction in permittivity with respect to Healthy.

**Table 4:** Binary classification, DD1, MLP hyper-parameters.

Training–Validation (%)		60:40	70:30	80:20
Real–Imag	Neurons	6	24	32
	Learning rate	0.03	0.01	0.045
	Training fn.	S-CGB	S-CGB	S-CGB
	Loss fn.	MSE	MSE	SAE
Module	Neurons	32	32	10
	Learning rate	0.035	0.035	0.035
	Training fn.	CGB	CGB	S-CGB
	Loss fn.	MSE	MSE	SAE

**Table 5:** Binary classification, DD2, MLP hyper-parameters.

Training–Validation (%)		60:40	70:30	80:20
Real–Imag	Neurons	10	48	12
	Learning rate	0.02	0.035	0.045
	Training fn.	CGB	CGB	S-CGB
	Loss fn.	MSE	MSE	SAE
Module	Neurons	24	48	24
	Learning rate	0.03	0.025	0.035
	Training fn.	S-CGB	S-CGB	S-CGB
	Loss fn.	MSE	SAE	SAE

**Table 6:** Binary classification, validation phase, accuracy results in percentages.

Training–Validation (%)		60:40	70:30	80:20
DD1	Real–Imag	99.35	99.31	99.74
	Module	96.74	98.44	98.18
DD2	Real–Imag	99.74	98.26	98.44
	Module	97.92	99.48	98.18

**Table 7:** Binary classification, DD1, testing phase, results in percentages.

Training–Validation (%)		60:40	70:30	80:20
Real–Imag	accuracy	73.54	60.62	61.04
	precision	82.70	77.97	78.10
	recall	73.54	60.63	61.04
	f1-score	71.55	53.40	54.07
Module	accuracy	94.37	73.75	81.25
	precision	94.65	82.79	82.14
	recall	94.37	73.75	81.25
	f1-score	94.36	71.81	81.12

**Table 8:** Binary classification, DD2, testing phase, results in percentages.

Training–Validation (%)		60:40	70:30	80:20
Real–Imag	accuracy	60.00	73.33	72.08
	precision	77.78	82.16	82.00
	recall	60.00	73.33	72.08
	f1-score	52.38	71.29	69.72
Module	accuracy	83.33	92.71	80.00
	precision	86.84	93.34	85.44
	recall	83.33	92.71	80.00
	f1-score	82.93	92.68	79.20

**Table 9:** Multi-class classification, module configuration, MLP hyper-parameters.

Hyper-Param.	DD1, 60:40 (%)		DD2, 70:30 (%)	
	4 Classes	2 Classes	4 Classes	2 Classes
Neurons	32	2	24	2
Learning rate	0.045	0.03	0.02	0.045
Training fn.	CGB	S-CGB	S-CGB	S-CGB
Loss fn.	MSE	MSE	MSE	MSE

## Data Availability

The original contributions presented in this study are included in the article. The collected dataset used to train, validate and test the classifiers is available for download as Supplementary Materials. Further inquiries can be directed to the corresponding author.

## Supplementary Materials

The following supporting information can be downloaded at: https://www.mdpi.com/article/10.3390/s25092718/s1.

## Abbreviations

The following abbreviations are used in this manuscript:

AD	Alzheimer’s disease
ANN	Artificial neural networks
CGB	Conjugate gradient method
CSF	Cerebrospinal fluid
CT	Computed tomography
DD1	Data division one
DD2	Data division two
FN	False negative
FP	False positive
MIMO	Multiple input multiple output
ML	Machine learning
MLP	Multilayer perceptron
MRI	Magnetic resonance imaging
MSE	Mean squared error
MWS	Microwave sensing
PCA	Principal components analysis
PET	Positron emission tomography
cPLA	Carbon-loaded Polylactic acid
SAE	Sum absolute error
S-CGB	Scaled conjugate gradient method
SVM	Support vector machine
TN	True negative
TP	True positive
VNA	Vector network analyzer
